# Hypoxia briefly increases diuresis but reduces plasma volume by fluid redistribution in women

**DOI:** 10.1152/ajpheart.00394.2022

**Published:** 2022-10-21

**Authors:** Johanna Roche, Peter Rasmussen, Hannes Gatterer, Giulia Roveri, Rachel Turner, Gerrit van Hall, Marc Maillard, Anna Walzl, Michael Kob, Giacomo Strapazzon, Jens Peter Goetze, Simon Thomas Schäfer, Tobias Kammerer, Elie Nader, Philippe Connes, Mélanie Robert, Thomas Mueller, Eric Feraille, Christoph Siebenmann

**Affiliations:** ^1^Institute of Mountain Emergency Medicine, Eurac Research, Bolzano, Italy; ^2^H. Lundbeck A/S, Copenhagen, Denmark; ^3^Department of Clinical Biochemistry, Rigshospitalet, University of Copenhagen, Copenhagen, Denmark; ^4^Department of Biomedical Sciences, Faculty of Health and Medical Sciences, University of Copenhagen, Copenhagen, Denmark; ^5^Clinical Metabolomics Core Facility, Rigshospitalet, University of Copenhagen, Denmark; ^6^Service of Nephrology, University Hospital of Lausanne, Lausanne, Switzerland; ^7^Department of Anesthesiology, LMU Klinikum, Ludwig-Maximilians-University München, Munich, Germany; ^8^Division of Clinical Nutrition, Bolzano Regional Hospital, Bolzano, Italy; ^9^Department for Anesthesiology and Intensive Care Medicine, Faculty of Medicine and University Hospital Cologne, University of Cologne, Cologne, Germany; ^10^Laboratory LIBM EA7424, Vascular Biology and Red Blood Cell Team, University of Lyon, Lyon, France; ^11^Department of Clinical Pathology, Hospital of Bolzano, Bolzano, Italy; ^12^Department of Laboratory Medicine, Hospital Voecklabruck, Voecklabruck, Austria; ^13^National Center of Competence in Research Kidney Control of Homeostasis (Kidney.CH), Zurich, Switzerland; ^14^Department of Cellular Physiology and Metabolism, University of Geneva, Geneva, Switzerland

**Keywords:** acclimatization, altitude, female, inflammation, total body water

## Abstract

We have recently reported that hypobaric hypoxia (HH) reduces plasma volume (PV) in men by decreasing total circulating plasma protein (TCPP). Here, we investigated whether this applies to women and whether an inflammatory response and/or endothelial glycocalyx shedding could facilitate the TCCP reduction. We further investigated whether acute HH induces a short-lived diuretic response that was overlooked in our recent study, where only 24-h urine volumes were evaluated. In a strictly controlled crossover protocol, 12 women underwent two 4-day sojourns in a hypobaric chamber: one in normoxia (NX) and one in HH equivalent to 3,500-m altitude. PV, urine output, TCPP, and markers for inflammation and glycocalyx shedding were repeatedly measured. Total body water (TBW) was determined pre- and postsojourns by deuterium dilution. PV was reduced after 12 h of HH and thereafter remained 230–330 mL lower than in NX (*P* < 0.0001). Urine flow was 45% higher in HH than in NX throughout the first 6 h (*P* = 0.01) but lower during the second half of the first day (*P* < 0.001). Twenty-four-hour urine volumes (*P* ≥ 0.37) and TBW (*P* ≥ 0.14) were not different between the sojourns. TCPP was lower in HH than in NX at the same time points as PV (*P* < 0.001), but inflammatory or glycocalyx shedding markers were not consistently increased. As in men, and despite initially increased diuresis, HH-induced PV contraction in women is driven by a loss of TCPP and ensuing fluid redistribution, rather than by fluid loss. The mechanism underlying the TCPP reduction remains unclear but does not seem to involve inflammation or glycocalyx shedding.

**NEW & NOTEWORTHY** This study is the first to investigate the mechanisms underlying plasma volume (PV) contraction in response to hypoxia in women while strictly controlling for confounders. PV contraction in women has a similar time course and magnitude as in men and is driven by the same mechanism, namely, oncotically driven redistribution rather than loss of fluid. We further report that hypoxia facilitates an increase in diuresis, that is, however, short-lived and of little relevance for PV regulation.

## INTRODUCTION

Extended hypoxic exposure facilitates a reduction in plasma volume (PV) that increases hemoglobin concentration ([Hb]) and thus restores arterial O_2_ content ($\text{C}{\text{a}}_{{\text{O}}_{2}}$) despite persistent reductions in arterial O_2_ saturation (1). We have recently reported this PV contraction to reflect a fluid shift from the intra- to the extravascular compartment, oncotically driven by a reduction in total circulating plasma protein (TCPP) (2). These results, however, prompted further questions.

First, as our previous study included only males, it remained unclear whether the results also apply to females. In fact, some studies have indicated hypoxia-induced PV contraction to be slower and less pronounced in women (3, 4). Confounders that can affect body fluid, such as physical activity (5) or the menstrual cycle (6), were, however, not controlled for in these studies. A blunted PV response to hypoxia in women therefore warrants confirmation in a strictly controlled setting, along with an evaluation of the underlying mechanisms.

Second, although the increased diuretic fluid loss was widely considered the main mechanism for hypoxia-induced PV contraction (1, 7, 8), we did not observe any effects of hypoxia on 24-h urine volumes or total body water (TBW) (2). This finding conflicted with well-controlled studies, which reported acute hypoxia (<12 h) to increase diuresis (7, 9, 10). We speculated that the first hours of hypoxia facilitate an increase in diuresis that is followed by a compensatory reduction, thus preserving 24-h urine volume, but this requires confirmation.

Third, the mechanism underlying the TCPP reduction in hypoxia remained unclear. Although an inflammatory response to hypoxia could increase the vascular permeability for albumin (11, 12), the proinflammatory effect of hypoxia is controversial as it has been observed by some (13–17) but not by others (18, 19). As previous studies have included only a few measurement time points, it is further unclear whether the time course of a potential inflammatory response aligns with that of PV contraction. Recent studies (20, 21) have further indicated that hypoxia accelerates the shedding of the endothelial glycocalyx, which could increase vascular permeability (22). Nevertheless, these studies included more severe hypoxia and, in one case (21), a strenuous 2-day ascent that could have affected the integrity of the glycocalyx independently of hypoxia (23). As such, it remains unclear whether these findings apply to our setting.

Accordingly, this study investigated the effects of hypobaric hypoxia (HH) on PV in healthy female lowlanders, while controlling for menstrual cycle variations. As previously published (2), subjects completed two 4-day sojourns under strictly controlled conditions, one in normoxia (NX) and one in HH, and the effects of the two exposures were compared. We hypothesized that *1*) the HH-induced PV contraction is slower and smaller than recently reported in men, *2*) TCPP decreases in HH, *3*) TBW is not affected by HH, *4*) HH facilitates an acute increase in diuresis but no change in 24-h urine volume, and *5*) the reduction in TCPP is accompanied by an increase in inflammatory and/or endothelial glycocalyx shedding markers.

## MATERIALS AND METHODS

This study was performed in accordance with the Declaration of Helsinki and approved by the Ethics Committee of the Bolzano Hospital, Italy (No. 70-2019). Twelve healthy, nonsmoking, female lowlanders (24.0 ± 4.2 yr, 59.6 ± 7.4 kg, 1.68 ± 0.08 m) without a history of high-altitude illness were enrolled as subjects, after providing written informed consent. An inclusion criterion was that they used either a combined oral (*n* = 11) or progestin intrauterine (*n* = 1) contraception. Since the study was conducted during the COVID-19 pandemic, a further inclusion criterion was that they were either fully vaccinated or had recovered from an infection. Subjects always underwent a COVID-19 PCR test before entering, and a rapid antigen test before leaving our facilities, all of which were negative.

### Protocol

Subjects completed two 4-day sojourns in a hypobaric chamber (263 m; terraXcube, Eurac Research, Bolzano, Italy): one in NX, where barometric pressure was not modified, and one in HH, where barometric pressure was reduced to 493.5 mmHg (corresponding to ∼3,500-m altitude). Six subjects started with the NX, and the other six with the HH sojourn. Group allocation was based on the contraception calendar of the subjects so that study attendance occurred during the active phase of pill consumption and in the absence of menstruation for the progestin user. The onsets of the sojourns were separated by 4 wk so that they occurred at identical points of the subject’s contraception calendar.

Subjects reported to our facilities the evening preceding the sojourns and spent the night there. At 06:00 the next morning, they entered the chamber, which was then decompressed (for the HH sojourn) at a rate simulating an ascent of 2 m·s^−1^. Four days before and throughout the sojourns, subjects adhered to the same standardized diet consisting of three main meals (breakfast, 08:30; lunch, 13:00; and dinner, 19:00) and three snacks (10:30, 16:00, and 22:00), providing 1,970 kcal·day^−1^ (48.5% carbohydrates, 36.9% fat, and 14.6% protein) and 94.5 and 58.8 mmol·day^−1^ of Na^+^ and K^+^, respectively. Water intake was 2 L·day^−1^ divided into six equal portions that were ingested with the meals/snacks. For breakfast, subjects were allowed one cup of coffee or tea, the volume of which was deducted from the breakfast water portion. No alcohol was consumed. Daily step count was monitored throughout the 3 days preceding the first sojourn by a pedometer, and subjects were instructed to reproduce the average step count on each day of the NX (6,324 ± 2,264 steps·day^−1^) and HH sojourns (6,245 ± 2,242 steps·day^−1^; *P* = 0.07) by walking on a treadmill. No other endurance-type exercise was performed, but subjects were allowed light strength training and stretching. Throughout the days, subjects were free to work or engage in recreational activities but were confined to bed between 23:00 and 07:00 with the lights turned off. The temperature in the chamber was 22°C and the relative humidity was 30%.

**Figure 1.**
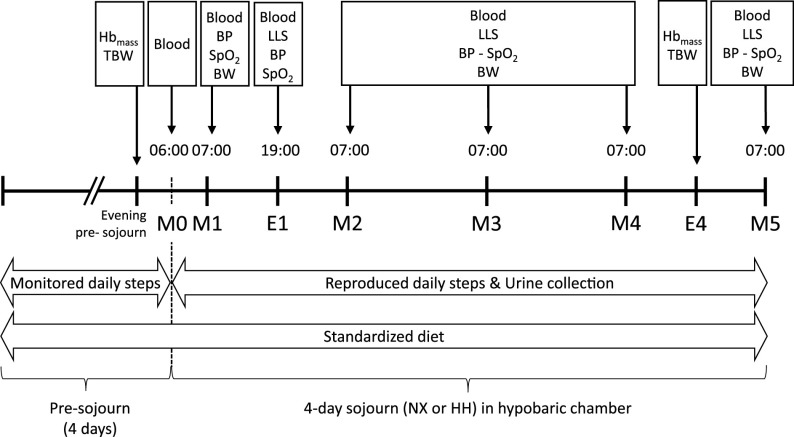
Measurements performed during the 4-day NX and HH sojourns. M0, morning presojourn; M1–M5, first to fifth morning of the sojourns, respectively; E1 and E4, first and fourth evening of the sojourns, respectively. BP, measurement of blood pressure by automatic sphygmomanometer; BW, body weight measurement; HH, hypobaric hypoxia; Hb_mass_, determination of total hemoglobin mass by carbon monoxide rebreathing; LLS, assessment of acute mountain sickness symptoms with the 1993 Lake Louise scoring system (24); NX, normoxia; $\text{S}{\text{p}}_{{\text{O}}_{2}}$, estimation of arterial oxyhemoglobin saturation by pulse oximetry; TBW, assessment of total body water by deuterium dilution.

Venous blood was collected in the morning before (M0) and after (M1) chamber entry at 06:00 and 07:00, respectively, the latter corresponding to ∼ 30 min of HH exposure in that sojourn. Further samples were obtained on the first evening at 19:00 (E1) and on every following morning at 07:00 (M2–M5) with total blood withdrawal remaining <100 mL per sojourn. Morning samples were obtained with subjects fasting and still in bed, whereas the evening sampling was preceded by 30 min of supine rest. At the same time points, arterial oxyhemoglobin saturation ($\text{S}{\text{p}}_{{\text{O}}_{2}}$) was assessed by pulse oximetry (Nonin 3150 Wrist Ox2, Plymouth, MN), and blood pressure and heart rate were assessed by an automatic sphygmomanometer (708-BT, Omron HealthCare, Kyoto, Japan). Symptoms of acute mountain sickness (AMS) were evaluated on E1 and M2–M5 using the self-reported Lake Louise Score (LLS) (24). Urine was continuously collected throughout both sojourns, and body weight was measured every morning after the first voiding. Total hemoglobin mass (Hb_mass_) and total body water (TBW) were measured on the evening preceding and on the last evening (E4) of the sojourns. The protocol is illustrated in Fig. 1.

### Blood Analyses

[Hb] as well as plasma Cl^−^, K^+^, and Na^+^ concentrations (ABL90 FLEX, Radiometer, Copenhagen, Denmark) and hematocrit (Hct) (micro-method, 4 min at 13,680 *g*) were measured in blood collected in heparinized syringes. The following variables were measured by blinded investigators in plasma from blood collected in anticoagulant vacutainers and centrifugated for 15 min at 1,600 *g* and 4°C: creatinine (Cr) and plasma protein concentration (PPC) were assessed by colorimetric assays (CREJ2, and TP2 Cobas, Roche Diagnostics, Basel, Switzerland). TCPP was calculated as PPC × PV, and $\text{C}{\text{a}}_{{\text{O}}_{2}}$ as 1.34 × [Hb] × $\text{S}{\text{p}}_{{\text{O}}_{2}}$/100 (25). Estrogen and progesterone concentrations were determined (on M0 and M5 only) by chemiluminescent immunoassay (Cobas, Roche Diagnostics). Plasma renin activity and aldosterone concentration were measured by radioimmunoassay. Kryptor PLUS automated platforms (Thermo Fisher Scientific, Waltham, MA) were used to measure plasma concentrations of copeptin and midregional proANP (MR-proANP), which served as proxies for antidiuretic hormone (ADH) (26) and atrial natriuretic peptide (ANP) (27), respectively. High-sensitivity C-reactive protein (hs-CRP) and interleukin-6 (IL-6) concentrations were assessed by immunoturbidimetric assay (Cobas 8000/c702, Roche Germany Holding, Grenzach, Germany) and electrochemiluminescence immunoassay (Cobas 8000/e801, Roche Germany Holding), respectively. For evaluation of endothelial glycocalyx shedding, syndecan-1 (ELISA kit, Diaclone SAS, Besançon, France), heparan sulfate (ELISA kit, Wuhan Fine Biotech, Wuhan, PR China), and hyaluronan concentrations (ELISA kit, Echelon Biosciences, Salt Lake City, UT) were determined on M0, M2, and M4.

### Hemoglobin Mass and Intravascular Volumes

Hb_mass_ was determined by carbon monoxide (CO) rebreathing as specified (28). Briefly, after 20 min of supine rest, the subject breathed O_2_ through a mouthpiece for 4 min. Thereafter, they were switched via a sliding valve to an O_2_-filled rebreathing circuit containing a 6-L external lung and a CO_2_ scrubber. After a venous blood collection, a CO dose of ∼1.2 mL·kg body wt^−1^ was injected into the rebreathing circuit. After 10 min of rebreathing, a second blood sample was collected. Furthermore, the absorbed volume of CO was determined by measuring the CO volume remaining in the rebreathing circuit and subtracting it from the injected volume. The blood samples were analyzed in quadruplicate by a hemoximeter (ABL90 FLEX), and Hb_mass_ was calculated from the absorbed CO volume and the resulting increase in carboxyhemoglobin (28). Intravascular volumes for M0–M5 and E1 were derived by integration of Hct and [Hb] determined at the respective time points as *1*) red blood cell volume (RBCV) = Hb_mass_ × Hct/[Hb], *2*) blood volume (BV) = RBCV/Hct, and *3*) PV = BV – RBCV. As previously published, Hb_mass_ measured on the evening preceding the study was used for the calculation of intravascular volumes on M0, M1, and E1, Hb_mass_ measured on E4 for calculation on M4 and M5, and the average of the two Hb_mass_ values for calculation on M2 and M3 (2).

### Total Body Water

At 2230 (∼30 min after the last food/water ingestion and ∼20 min after tooth brushing), subjects voided, whereafter a saliva sample was collected. Subsequently, ∼4 g (the exact weight was recorded for each subject) of deuterium oxide (D_2_O, 99.88%, Cambridge Isotope Laboratories, Tewksbury, MA), diluted in 70 mL of tap water, were ingested. The bottle was then rinsed with 130 mL of tap water, which was also ingested (to preserve the daily water intake, the water portion preceding the D_2_O ingestion was reduced by 200 mL). At 0830 on the next morning, a second saliva sample was collected. Saliva isotope enrichment relative to standard mean ocean water was determined in quadruplicate by isotope-ratio mass spectrometry (Gasbench II, Conflo IV, Delta V advantage, Thermo Scientific, Bremen, Germany). TBW was calculated as the D_2_O dilution space divided by 1.04 to correct for nonaqueous exchange using the formula by Schoeller et al. (29).

### Urine Volumes and Renal Function

Urine collection started at 07:00 on the first morning of the sojourns, after the first voiding. Volumes produced during the first 6 and 12 h of the sojourns were determined, with subjects voiding immediately before these determinations. Thereafter, 24-h urine volumes were measured throughout the sojourns, with the sampling interval always starting in the morning after the first voiding and ending with the first voiding on the next day. In 24-h urine, Cl^−^, K^+^, and Na^+^ concentrations were determined by an ion-selective electrode (Cobas, Roche Diagnostics), Cr and protein concentrations by colorimetric assays (CREJ2, and TP2 Cobas, Roche Diagnostics), and syndecan-1, heparan sulfate, and hyaluronan by the same technique as for plasma.

Average glomerular filtration rate (GFR) for each day was estimated based on Cr clearance as GFR = ([Cr]_urine_ × urine flow rate)/[Cr]_plasma_, where [Cr]_plasma_ was the average of the plasma Cr concentrations measured at the beginning and end of the 24-h urine collection, respectively, and urine flow rate of the 24-h urine volume (in mL) divided by 1,440 min. Daily urinary excretion of protein, Na^+^, K^+^, and Cl^−^ was calculated by multiplying the urine solute concentration with the 24-h urine volume, and tubular reabsorption of the solutes as GFR × average plasma solute concentration – solute excretion·min^−1^. H_2_O reabsorption was calculated as GFR – urine flow rate.

### Statistical Analyses

Statistical analyses were performed using SAS 9.4 (SAS Institute, Cary, NC). Data are presented as means ± SD, unless stated otherwise. We assessed potential preexposure differences between NX and HH using paired *t* tests or Wilcoxon signed-rank tests, as appropriate. Data from M0 to M5 were analyzed by mixed model for repeated measurements (MMRM) with fixed factors of “altitude” (NX vs. HH), order of sojourns (NX-HH vs. HH-NX), measurement time points, and with random effect of the subject. To isolate the effect of HH from any other effects of the chamber confinement, pairwise comparisons of matched time points were performed as contrasts within the MMRM model and adjusted for multiplicity by Sidak’s method. Changes over time within a given sojourn were not evaluated as the study was not designed for this and to preserve statistical power. We did not impute missing data, as MMRM analysis controls type I error and minimizes bias. Values below the detection threshold were estimated as half that threshold.

## RESULTS

One subject left the study for personal reasons after completing the HH sojourn only. Her data were included in the statistical analyses and written results, whereas in the figures, they are presented as individual data points but not included in average values. Another subject completed the NX sojourn but left the chamber on the last evening of the HH sojourn for health problems that were, to the best of our knowledge, unrelated to the study interventions. Her value for TBW at that time point and all her data for M5 in HH are hence missing.

In HH, the LLS indicated mild AMS (LLS = 3 to 4) in four subjects on E1, and in another subject on M2 and M4. However, LLS ≥ 3 were reported in NX as well, namely, on E1 (*n* = 2) and M2 (*n* = 1), and the LLS was not different between the sojourns at any time point (all *P* ≥ 0.44). $\text{S}{\text{p}}_{{\text{O}}_{2}}$ was lower in HH than in NX from M1 (86.8 ± 2.2 vs. 97.4 ± 0.8%, *P* < 0.0001) to M5 (89.7 ± 1.9 vs. 97.8 ± 0.8%, *P* < 0.0001), whereas $\text{C}{\text{a}}_{{\text{O}}_{2}}$ was lower in HH from M1 to M3 (all *P* ≤ 0.01) but not different from NX on M4 (*P* = 0.70) and M5 (*P* = 0.32).

### Sex Hormones

Plasma estrogen concentrations were not different between NX and HH on M0 (9.46 ± 14.78 vs. 16.95 ± 17.09 pg·mL^−1^, *P* = 0.15) and M5 (13.14 ± 19.20 vs. 9.44 ± 14.15 pg·mL^−1^, *P* > 0.99), and this was also the case for progesterone concentrations (M0, 0.25 ± 0.15 vs. 0.32 ± 0.11 ng·mL^−1^, *P* = 0.12; and M5, 0.25 ± 0.09 vs. 0.27 ± 0.16 ng·mL^−1^, *P* = 0.88). No correlations (Spearman, all *P* > 0.05) were found between individual progesterone or estrogen concentrations (average between M0 and M5 values of the HH sojourn) and the magnitude of the HH-induced PV contraction.

### Intravascular Volumes

**Figure 2.**
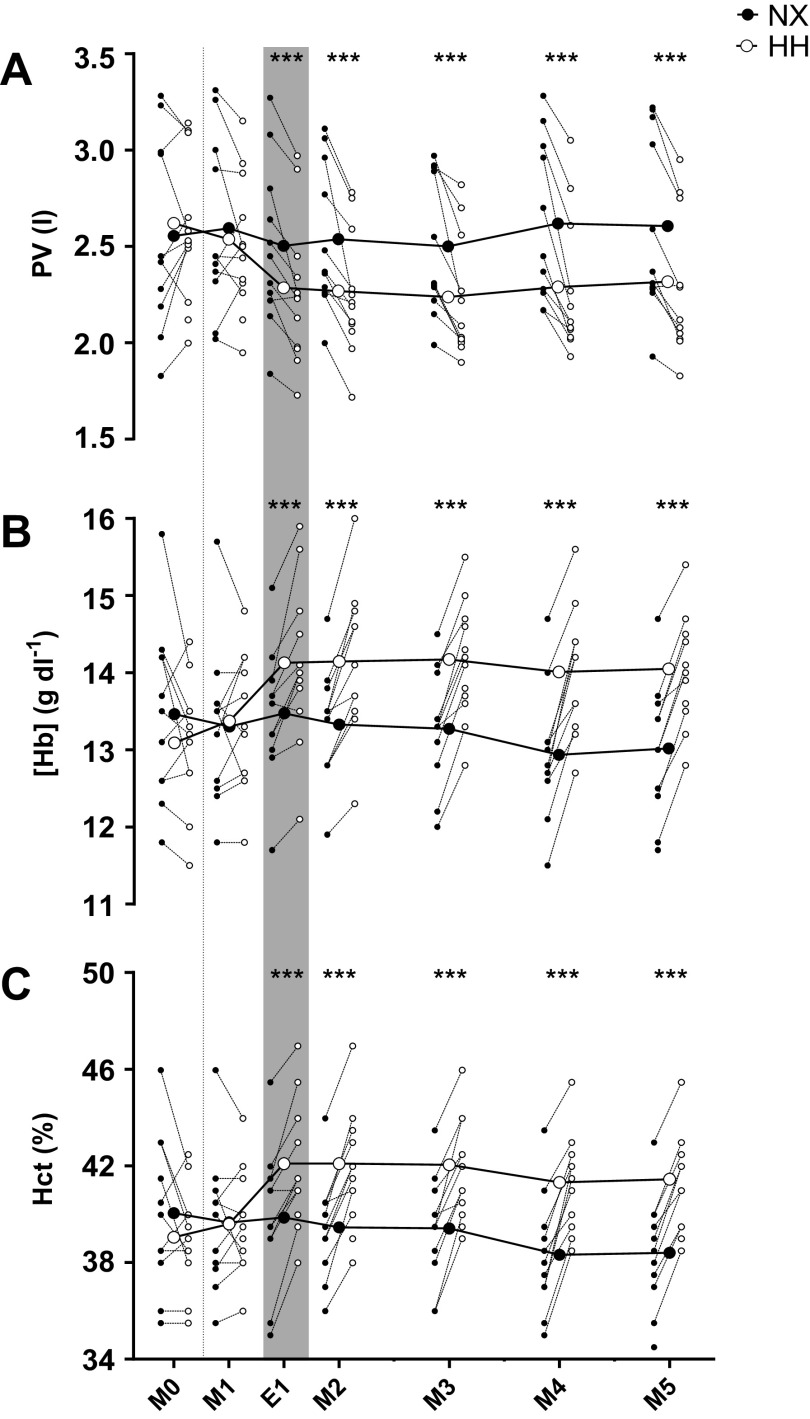
Effects of hypoxia on plasma volume. Plasma volume (PV; *A*), venous hemoglobin concentration ([Hb]; *B*), and hematocrit (Hct; *C*) throughout 4-day sojourns in normoxia (NX) and hypobaric hypoxia (HH). M0, morning presojourn; M1–M5, first to fifth morning of the sojourns, respectively; E1, first evening of the sojourns. Large symbols represent means; small symbols represent individual data. ***P < 0.001 for time point comparisons between NX and HH by mixed model for repeated-measurement analyses. Dotted line marks the onset of the sojourns, and gray shaded area marks measurements performed in the evening.

Hb_mass_ was not different between NX and HH both before (568 ± 75 vs. 559 ± 79 g, *P* = 0.99) and at the end of the sojourns (548 ± 82 vs. 547 ± 79 g, *P* = 0.51). PV (Fig. 2*A*) did not differ between NX and HH at M0 (*P* = 0.44) and M1 (*P* = 0.19) but was 230–330 mL lower in HH at all later time points (all *P* < 0.0001). As a result, [Hb] and Hct (Fig. 2, *B* and *C*) were higher, and BV was lower in HH than NX from E1 to M5 (all *P* < 0.0001).

### Body Fluid Regulation

**Figure 3.**
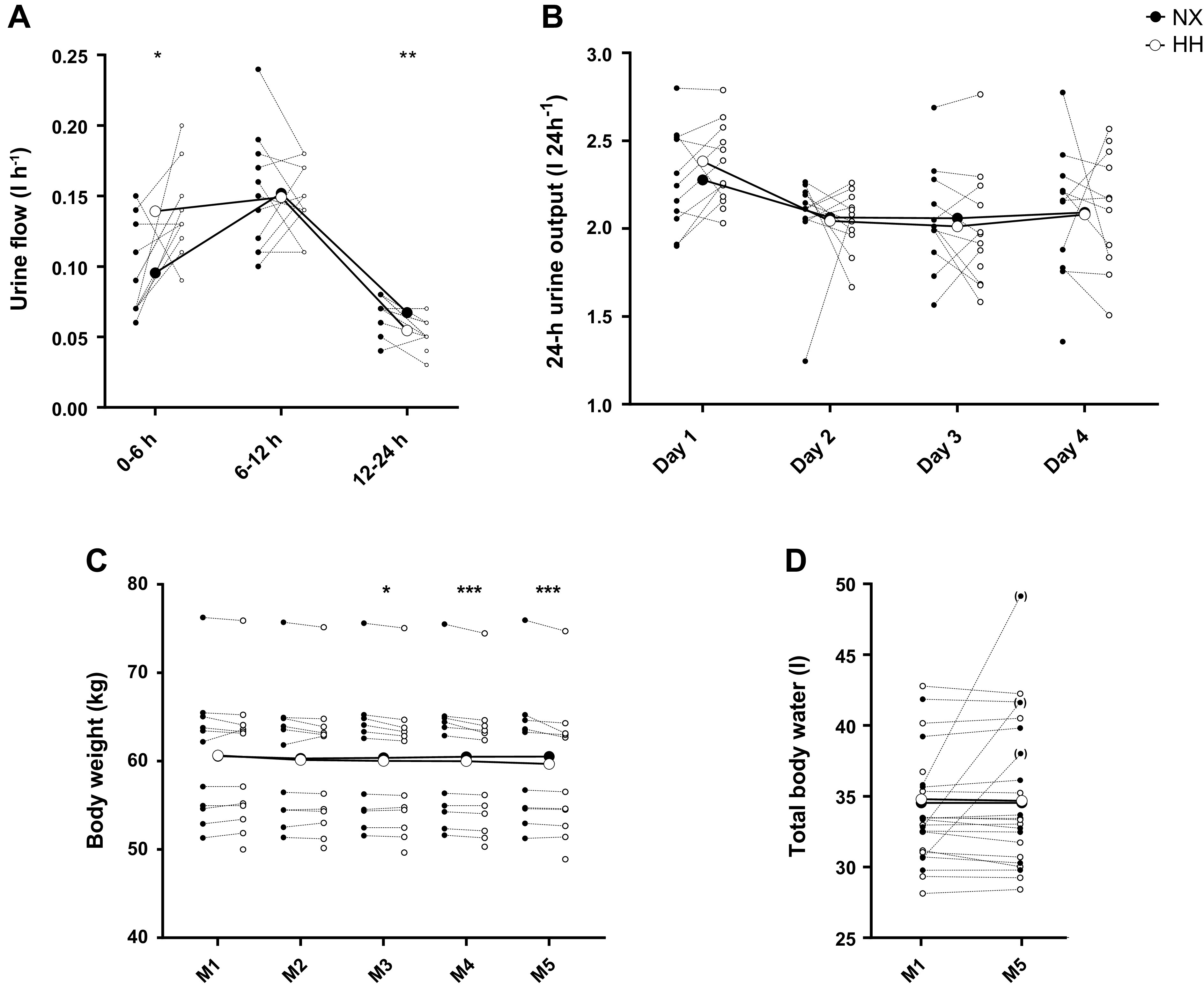
Effects of hypoxia on body fluid regulation. Urine flow measured over the first 24 h of a 4-day sojourn in normoxia (NX) and hypobaric hypoxia (HH; *A*), 24-h urine output (*B*), body weight (*C*), and total body water (*D*) throughout the sojourns. M1–M5, first to fifth morning of the sojourns, respectively. Large symbols represent means; small symbols represent individual data. *P < 0.05; **P < 0.01; ***P < 0.001 for time point comparisons between NX and HH by mixed model for repeated-measurement analyses. Individual data points in brackets represent outliers that were not included in the analyses.

Urine flow (Fig. 3*A*) was 45% higher in HH than in NX throughout the first 6 h (*P* = 0.01) of the sojourns, not different between sojourns throughout the 6- to 12-h interval (*P* = 0.99) and lower in HH throughout the 12- to 24-h interval (*P* < 0.01). Consequentially, none of the 24-h urine volumes (Fig. 3*B*) differed between the sojourns (all *P* ≥ 0.37). Body weight (Fig. 3*C*) did not differ between the sojourns on M1 and M2 but was 0.3–0.5 kg lower in HH than NX from M3 to M5 (all *P* ≤ 0.01). TBW (Fig. 3*D*) was not different between NX and HH both on the evenings preceding the sojourns (*P* = 0.14) and on E4 (*P* = 0.19). For unknown reasons, analysis of three saliva samples collected in NX on E4 yielded D_2_O values that resulted in an implausibly high TBW; these values were not included in the analyses but are shown in the figure as outliers.

### Plasma and Urine Proteins

**Figure 4.**
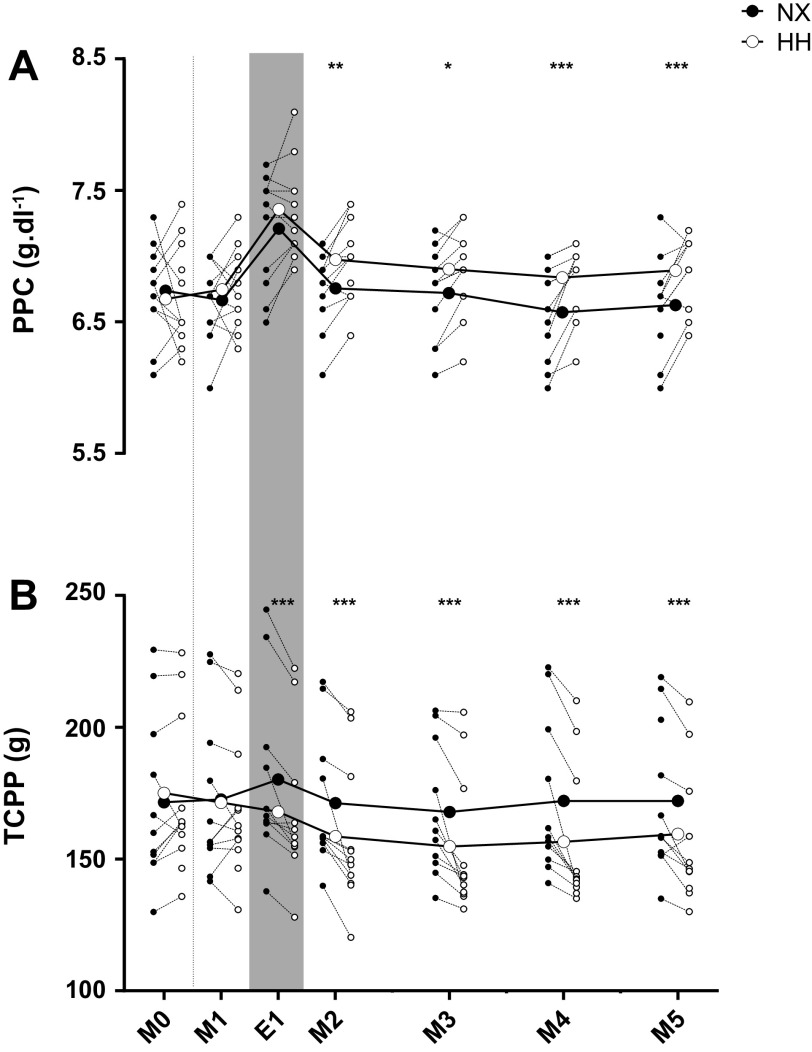
Effects of hypoxia on plasma proteins. Plasma protein concentration (PPC; *A*) and total circulating plasma protein (TCPP; *B*) throughout 4-day sojourns in normoxia (NX) and hypobaric hypoxia (HH). M0, morning presojourn; M1–M5, first to fifth morning of the sojourns, respectively; E1, first evening of the sojourns. Large symbols represent means; small symbols represent individual data; *P < 0.05; **P < 0.01; ***P < 0.001 for time point comparisons between NX and HH by mixed model for repeated-measurement analyses. Dotted line marks the onset of the sojourns, and the gray shaded area marks measurements performed in the evening. One data point on E1 in HH (PPC = 3.2 g·dL^−1^, corresponding to a TCPP of 63 g) was considered an outlier, excluded from analyses and not shown in the plots.

PPC (Fig. 4*A*) did not differ between the sojourns on M0 and M1 (all *P* ≥ 0.37), tended to be higher in HH on E1 (*P* = 0.06), and was higher in HH from M2 until the end of the sojourns (all *P* ≤ 0.01). TCPP (Fig. 4*B*) was not different between sojourns on M0 and on M1 (both *P* ≥ 0.28), but lower in HH from E1 until the end of the sojourns (all *P* < 0.0001). An apparent measurement error occurred for one value on E1 in HH (PPC= 3.2 g·dL^−1^, corresponding to a TCPP of 63 g). These data were excluded from analyses and not shown in the plots as they would have considerably expanded the *y*-axis.

Daily urinary protein excretion was not different between the sojourns at any time point (all *P* ≥ 0.22), resulting in a total protein excretion of 460 ± 300 and 390 ± 177 mg (*P* = 0.46) throughout the NX and HH sojourns, respectively.

### Markers of Inflammation and Endothelial Glycocalyx Shedding

**Figure 5.**
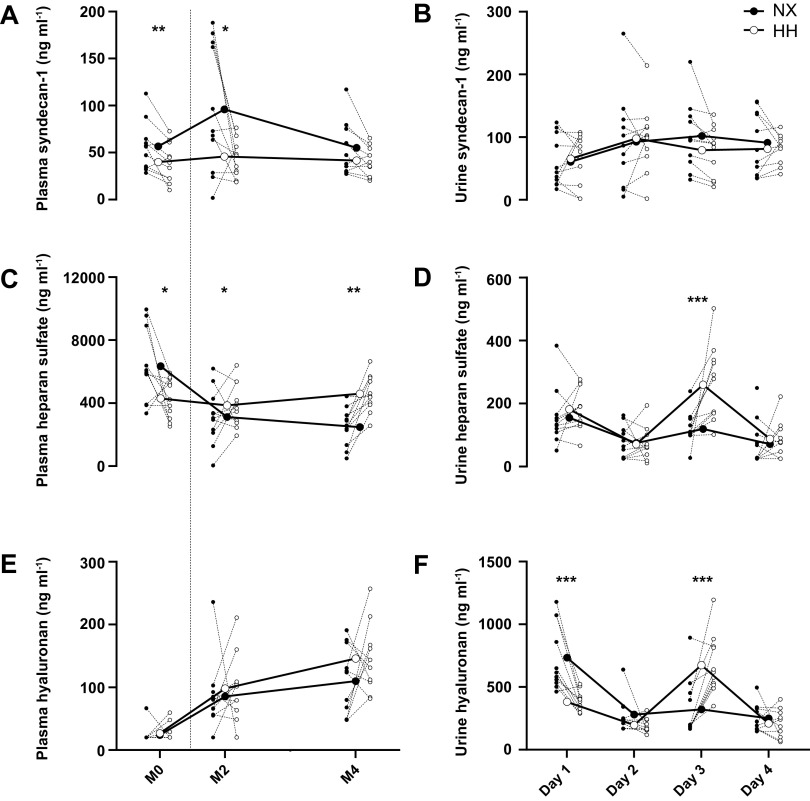
Effects of hypoxia on endothelial glycocalyx shedding. Plasma and 24-h urine concentrations of syndecan-1 (*A* and *B*), heparan sulfate (*C* and *D*), and hyaluronan (*E* and *F*) throughout 4*-*day sojourns in normoxia (NX) and hypobaric hypoxia (HH). M0, morning presojourn; M2–M4, second to fourth morning of the sojourns, respectively. Large symbols represent means; small symbols represent individual data. *P < 0.05; **P < 0.01; ***P < 0.001 for time point comparisons between NX and HH by mixed model for repeated-measurement analyses. Dotted line marks the onset of the sojourns. *E*: 90.9 and 72.7% of the samples collected in NX and HH on M0 were below the detection threshold and estimated as half this threshold (20 ng·mL^−1^).

Inflammatory markers are presented in Table 1. Hs-CRP concentrations remained below the detection threshold (0.1 mg·dL^−1^) for 55.8 and 30.5% of the samples collected in NX and HH, respectively. Similarly, IL-6 concentrations were below the detection threshold (1.5 pg·mL^−1^) for 74.0 and 62.2% of the samples collected in NX and HH, respectively. Given this large fraction of values below the detection threshold, it did not appear justified to perform statistical analyses on these variables. Plasma syndecan-1 concentrations (Fig. 5*A*) were lower in HH than in NX on M0 (*P* < 0.01) and M2 (*P* = 0.01) but not different between the sojourns on M4 (*P* = 1.00), whereas urine syndecan-1 concentrations (Fig. 5*B*) did not differ between the sojourns on any day (all *P* ≥ 0.18). Plasma heparan sulfate concentrations (Fig. 5*C*) were lower in HH than in NX on M0 (*P* = 0.04) but higher in HH than in NX on M2 (*P* = 0.04) and M4 (*P* < 0.001). Urine heparan sulfate concentrations (Fig. 5*D*) were higher in HH than in NX on *day 3* (*P* < 0.001) but did not differ between the sojourns on any other day (all *P* ≥ 0.84). Plasma hyaluronan concentrations (Fig. 5*E*) were below the detection threshold (40 ng·mL^−1^) on M0 for 90.9 and 72.7% of the samples collected in NX and HH, respectively, and did not differ between the sojourns at any time points (all *P* ≥ 0.23). Urine hyaluronan concentrations (Fig. 5*F*) were lower in HH than in NX on *day 1* (*P* < 0.0001), higher on *day 3* (*P* < 0.0001), and not different between the sojourns on *days 2* and *4* (both *P* ≥ 0.53).

**Table 1.** Markers of inflammation during two 4-day sojourns in NX and HH

	M0	M1	E1	M2	M3	M4	M5
High-sensitivity C-reactive protein, mg·dL^−1^							
NX	0.10 [0.05–0.20]	0.10 [0.05–0.20]	0.10 [0.05–0.10]	0.05 [0.05–0.10]	0.05 [0.05–0.10]	0.05 [0.05–0.10]	0.05 [0.05–0.20]
Values < detection threshold*	5/11	5/11	5/11	6/11	8/11	7/11	7/11
HH	0.10 [0.05–0.20]	0.10 [0.05–0.20]	0.10 [0.05–0.20]	0.10 [0.06–0.20]	0.10 [0.05–0.20]	0.10 [0.05–0.20]	0.10 [0.05–0.20]
Values < detection threshold*	4/12	3/11	4/12	3/12	4/12	4/12	3/11
Interleukin-6, pg·mL^−1^							
NX	1.60 [0.75–2.6]	1.80 [0.75–4.60]	1.50 [0.75–1.70]	0.75 [0.75–1.70]	0.75 [0.75–0.75]	0.75 [0.75–0.75]	0.75 [0.75–0.75]
Values < detection threshold*	5/11	5/11	5/11	8/11	11/11	11/11	9/11
HH	0.75 [0.75–1.78]	0.75 [0.75–1.90]	1.23 [0.75–2.00]	0.75 [0.75–1.70]	0.75 [0.75–1.73]	0.75 [0.75–1.39]	0.75 [0.75–1.80]
Values < detection threshold*	7/12	6/11	6/12	8/12	8/12	9/12	7/11

Values are medians [25–75% interquartile ranges]. *Values below detection threshold were estimated as half that threshold (i.e., 0.05 mg·dL^−1^ for high-sensitivity C-reactive protein and 0.75 pg·mL^−1^ for interleukin-6). The number of values below the detection threshold is provided for each time point. NX, normoxia; HH, hypobaric hypoxia; M0, morning presojourn; M1–M5, first to fifth morning of the sojourns, respectively; E1, first evening of the sojourns.

### Volume-Regulating Hormones

**Figure 6.**
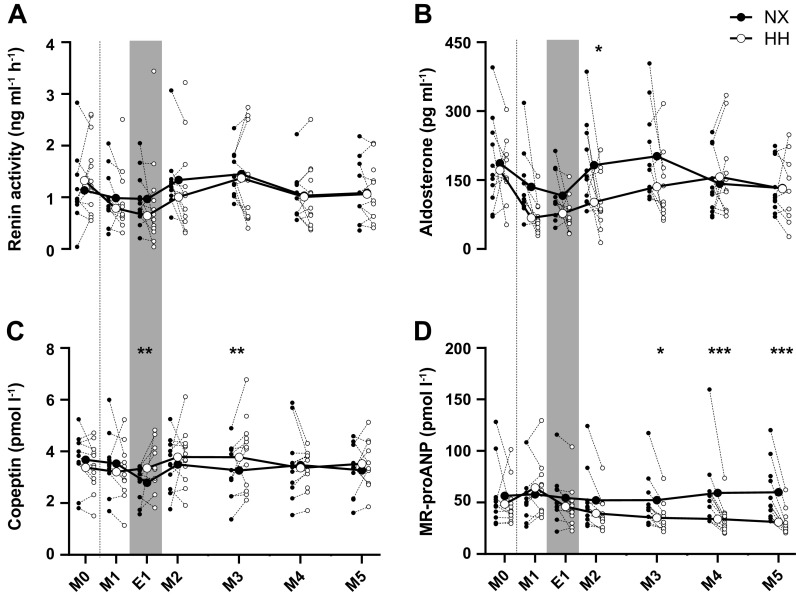
Effects of hypoxia on volume-regulating hormones. Renin activity (*A*), aldosterone (*B*), copeptin (*C*), and midregional proatrial natriuretic peptide (MR-proANP; *D*) concentrations in plasma throughout 4-day sojourns in normoxia (NX) and hypobaric hypoxia (HH). M0, morning presojourn; M1–M5, first to fifth morning of the sojourns, respectively; E1, first evening of the sojourns. Large symbols represent means; small symbols represent individual data. *P < 0.05; **P < 0.01; ***P < 0.001 for time point comparisons between NX and HH by mixed model for repeated-measurement analyses. Dotted line marks the onset of the sojourns, and gray shaded areas mark measurements performed in the evening. One subject exhibited very high aldosterone concentrations in HH that were considered as outliers, excluded from analyses, and not shown in the plots.

Plasma renin activity (Fig. 6*A*) did not differ between NX and HH at any time point (all *P* ≥ 0.11). Plasma aldosterone concentrations (Fig. 6*B*) tended to be lower in HH than in NX on M1 (*P* = 0.07) and M3 (*P* = 0.08) and were lower on M2 (*P* = 0.02) but did not differ from NX at any other time point (*P* > 0.67). One subject exhibited very high aldosterone concentrations (440–835 pg·mL^−1^) before and throughout the HH sojourn. These values were considered outliers, excluded from analyses, and not shown in the plot to avoid excessive expansion of the *y*-axis. Plasma copeptin concentrations (Fig. 6*C*) were higher in HH than in NX on E1 (*P* < 0.01) and M3 (*P* < 0.01) but not different between sojourns at any other time point (all *P* ≥ 0.16). Plasma MR-proANP concentrations (Fig. 6*D*) did not differ between NX and HH from M0 to M2 (all *P* ≥ 0.16) but were lower in HH from M3 (*P* = 0.01) to M5 (*P* < 0.0001).

### Renal Water and Electrolyte Handling

**Figure 7.**
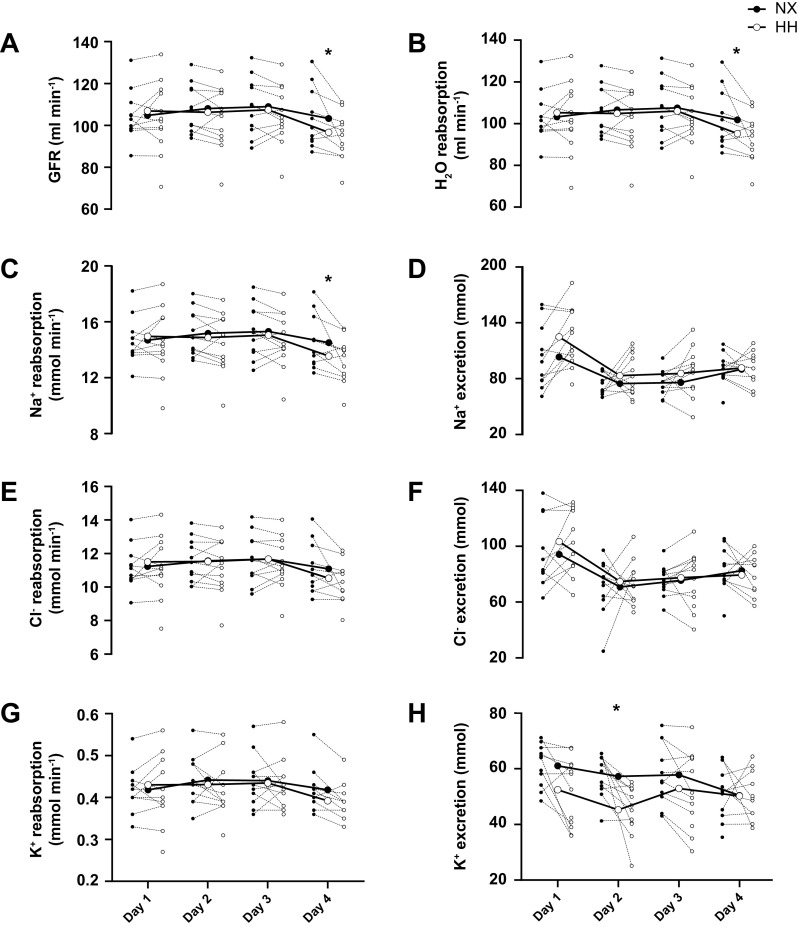
Effects of hypoxia on renal water and electrolyte handling. Glomerular filtration rate (GFR; *A*) and tubular H_2_O reabsorption (*B*), as well as tubular electrolyte reabsorption (*C*, *E*, and *G*) and excretion (*D*, *F*, and *H*) throughout 4-day sojourns in normoxia (NX) and hypobaric hypoxia (HH). Large symbols represent means; small symbols represent individual data. *P < 0.05 for time point comparisons between NX and HH by mixed model for repeated-measurement analyses.

GFR (Fig. 7*A*) and tubular H_2_O reabsorption (Fig. 7*B*) did not differ between NX and HH throughout the first 3 days (all *P* ≥ 0.47) but were both lower in HH on *day 4* (*P* = 0.015 and *P* = 0.014, respectively). Tubular Na^+^ reabsorption (Fig. 7*C*) was not different between NX and HH throughout the first 3 days (all *P* ≥ 0.37) but was lower in HH on *day 4* (*P* = 0.01), whereas Na^+^ excretion (Fig. 7*D*) did not differ between NX and HH at any time point (all *P* > 0.14). Tubular Cl^−^ reabsorption (Fig. 7*E*) was not different between sojourns at any time point (all *P* ≥ 0.29), although it tended to be lower in HH on *day 4* (*P* = 0.05), whereas Cl^−^ excretion (Fig. 7*F*) never differed between sojourns (*P* ≥ 0.85). Tubular K^+^ reabsorption (Fig. 7*G*) was not different between sojourns (all *P* ≥ 0.45), although it tended to be lower in HH on *day 4* (*P* = 0.07). K^+^ excretion (Fig. 7*H*) was lower in HH on *day 2* (*P* = 0.03) and not different between sojourns at any other time points (all *P* > 0.22).

## DISCUSSION

We investigated the regulation of the PV response to HH in healthy women. The PV reduction became statistically significant after 12 h of HH and reached a maximum of 330 mL, corresponding to 12.3%. In our recent study in men, the PV contraction became significant after 24 h (2) and reached a maximum of 340 mL, which corresponded to only 10.8%. Therefore, we reject the hypothesis of a slower and blunted PV contraction in women. In line with our hypotheses, however, PV contraction occurred in concert with a reduction in TCPP and unchanged TBW, supporting oncotically driven fluid redistribution from the intra- to the extravascular compartment. As further hypothesized, HH exposure led to an acute increase and ensuing reduction in urine flow but no changes in 24-h urine volumes. Finally, and contrary to our last hypothesis, markers of inflammation and endothelial glycocalyx shedding were not consistently increased by HH.

PV contraction is an almost universally observed response to hypoxic exposure (1), and we have recently identified oncotically driven fluid redistribution from the intra- to the extravascular compartment as the underlying mechanism in males (2). By replicating that study in a female cohort, we demonstrate here that not only the time course and magnitude of the PV contraction but also the underlying mechanisms are the same for both sexes. As in men, the PV contraction occurred in the absence of changes in 24-h urine output and TBW, confirming fluid redistribution rather than sensible or insensible water loss as the underlying mechanism, while the reduction in TCPP again supports that the fluid redistribution was oncotically driven. Of note, a raise in albumin and fibrinogen synthesis has been reported in subjects exposed to HH (30), suggesting that the amount of protein disappearing from the circulation could have been even greater than indicated by the TCPP reduction. What remains unclear is the fate of these proteins. Although Biollaz et al. (31) in their study recently reported ascent to 4,559-m altitude to induce mild microalbuminuria in subjects confined to bedrest, urinary protein excretion was identical between the sojourns in the current study. Transvascular protein escape has been suggested to occur as hypoxia-induced inflammation (11, 12) and/or endothelial glycocalyx shedding increase vascular permeability for albumin (21), but the unchanged inflammatory markers and inconsistent changes in markers for glycocalyx shedding in our study do not support these explanations. Regarding inflammation, we additionally performed an exploratory multiplex evaluation (Bio-Plex ProTMHuman Cyokine 17-plex Assay kit, Bio-Rad, Hercules, CA) of 16 inflammatory markers and found >90% of results to be below the detection threshold with no apparent difference between NX and HH (results not shown). The low and unchanged inflammatory status of our subjects could have reflected the absence of physical exercise (32, 33) and/or the relatively mild degree of hypoxia, since studies reporting an inflammatory response to hypoxia have commonly been conducted at much higher altitudes (13, 14, 16) or included hiking (15, 17). The low incidence of AMS could also have played a protective role, as inflammatory markers have been found to be increased with AMS (15, 16), although this is not a universal finding (13, 18). Regarding endothelial glycocalyx shedding, our results are somewhat more difficult to interpret. Although plasma heparan sulfate concentrations were indeed higher in HH than in NX on M2 and M4, hyaluronan and syndecan-1 concentrations were similar or even lower. Figure 5 further illustrates that the difference in heparan sulfate between the sojourns primarily reflected fluctuations throughout the NX, rather than the HH sojourn. In 24-h urine, the increased concentrations of heparan sulfate and hyaluronan on *day 3* are noteworthy, even though they were not accompanied by increased syndecan-1 concentrations. Nevertheless, it should be remembered that the HH-induced TCPP reduction primarily took place over the first 24 h of the HH sojourn, where urinary markers of glycocalyx shedding were similar to or even lower than in NX. Finally, the functional significance of all the observed differences in glycocalyx shedding markers between NX and HH is questionable, since all the plasma concentrations remained within previously published normal ranges (34, 35) and since it was recently postulated that only fivefold or higher increases constitute evidence for glycocalyx shedding (36).

Although the unchanged 24-h urine volumes in our previous study seemed to refute the broadly accepted paradigm that hypoxic exposure increases diuresis, it was difficult to explain why other well-controlled studies have found acute hypoxia to increase urine flow (7, 9, 10). Here, we provide an explanation for these apparently conflicting outcomes by demonstrating that acute HH induces a transient increase in urine flow, followed by a compensatory reduction that preserves 24-h urine volume. Loeppky et al. (10) in their study found indications for a similar biphasic response to a more severe HH, at least in subjects who tolerated the exposure well; however, in that study, water intake was adjusted to urine output, which made the interpretation more difficult than in the current setting. A short-lived diuresis increase within 1 h after arrival at 4,559 m altitude was also reported in a study by Biollaz et al. (31), although the authors attributed this phenomenon to the excitement associated with the passive ascent by helicopter. The acute increase in urine flow in HH could have been mediated by the reduction in circulating aldosterone that was observed on M1 (even though it did not quite reach statistical significance). Alternatively, it could have reflected renal excretion of bicarbonate compensating for the alkalosis resulting from the increased pulmonary ventilation in HH (9). Another explanation suggested by Swenson et al. (7), who found fluid-regulating hormone changes in acute hypoxia not to correlate with the concomitant increase in urine flow, is that activation of arterial chemoreceptors increases diuresis through a direct neuronal pathway or an unknown diuretic factor. Conversely, the reduction in urine flow after 12 h in HH could have been driven by the increase in ADH that was indicated by the elevated plasma copeptin concentration on E1. Whatever the mechanism, it is unlikely that the short-lived diuretic response contributed to PV contraction since it increased the urine volume accumulated over the first 6 h of HH by only 256 mL. Given the unrestricted fluid exchange between the intra- and extravascular compartment, this volume must have been drawn from the entire extracellular compartment, of which PV constitutes only ∼20%. It should further be considered that the ensuing reduction in urine output presumably restored any initial reductions in extracellular fluid volume. Taken together, although our data confirm that the diuretic response to hypoxia commonly referred to as “altitude diuresis” occurs even in the absence of confounding factors, it also reveals this phenomenon as short-lived and of little relevance for PV regulation.

The responses of the volume-regulating hormones to HH were also similar to those recently observed in men, consisting of a tendency for renin and aldosterone suppression throughout the first half and a reduction in MR-proANP release throughout the second half of the sojourn. The latter presumably reflected a decrease in central blood volume secondary to the PV contraction (37). However, one difference is that in women, we did not detect the marked increase in MR-proANP that occurred in men after 30 min of HH. As ANP release in acute hypoxia has been attributed to an increase in right ventricular afterload resulting from hypoxic pulmonary vasoconstriction (HPV) (38–40), the absence of a significant MR-proANP response could have reflected an attenuated HPV (41–43), even though a study (44) reported HPV to be greater in women than in men. Also, the effects of HH on renal water and electrolyte handling do not appear to differ between sexes: as in men, GFR was reduced in HH toward the end of the sojourn, likely because of a PV contraction-induced reduction in renal plasma flow (45), whereas the concomitant reduction in tubular water and sodium reabsorption presumably explains why this reduction in GFR did not translate into a lower urine output.

An observation that was not made in the male cohort (2) was that BW decreased in HH by ∼0.5 kg despite the controlled diet and maintained TBW. This weight loss could have reflected, at least in part, the raise in basal metabolic rate (BMR) that often (46–48), but not always (49), occurs during hypoxic exposure. Nevertheless, if the weight loss was indeed a consequence of a higher BMR, it is surprising that it did not occur in men, where hypoxia has been reported to facilitate a more pronounced and longer increase in BMR than in women (50).

Finally, we again found the incidence of AMS associated with rapid ascent to a simulated altitude of 3,500 m to be negligible (2), which seems in contrast with the literature (51). Of note, when using the revisited LLS that excludes the evaluation of sleep (52), the AMS incidence in our study was even lower. On one hand, this low incidence could have been attributable to the absence of strenuous exercise (53), adequate hydration (54), or an ascent in the early morning, which extended the acclimatization time preceding the first night, where AMS incidence usually peaks (51). On the other hand, it may have reflected that the diagnosis of AMS was not a primary aim of this study. Indeed, it has been recently discovered that studies explicitly focusing on AMS report markedly higher incidences, presumably as they increase participants’ expectations of experiencing the nonspecific symptoms that characterize AMS (55).

There are methodological aspects to consider. We investigated the isolated effects of HH, which, during high-altitude expeditions, may be confounded by changes in other environmental or behavioral factors. As previously published (2), we chose an exposure duration of 4 days since the major changes in PV occur during this period and a severity of HH that reduces PV (1) while not being associated with a high risk for severe AMS. The latter could have resulted in not only dropouts but also in water and salt homeostasis perturbation because of water retention and increased sodium reabsorption (10) or gastrointestinal symptoms (56).

We have used CO rebreathing to measure Hb_mass_ and inferred PV by integration of Hct and [Hb] (28). This approach has also been used in our previous study in men (2), thus allowing a comparison of results. Of note, in a previous study, CO rebreathing indicated a decrease in PV following 24 h of HH exposure that was not detected by the Evans blue dye dilution technique (57). Nevertheless, as Evans blue binds to albumin, an increased vascular permeability for proteins could have increased its dilution space, thus leading to an overestimation of PV in HH (57).

In conclusion, HH-induced PV contraction in women has a similar time course and magnitude as in men and is driven by the same mechanism, namely, an oncotically driven fluid shift from the intra- to the extravascular compartment. We furthermore confirm the existence of an “altitude diuresis,” that is, however, short-lived and seems irrelevant for PV regulation. Finally, as we find no support for the theory that hypoxia-induced inflammation and/or endothelial glycocalyx shedding increase vascular permeability for plasma proteins, the mechanism underlying the TCPP reduction in hypoxia remains unclear.

## DATA AVAILABILITY

The data that support the findings of this study are available from the corresponding author upon reasonable request.

## GRANTS

This work was funded by Swiss National Centre of Competence in Research (NCCR) Kidney Control of Homeostasis Grant N-403-03-51 (Kidney.ch) and the Herbert N. Hultgren Grant (Wilderness Medical Society, USA).

## DISCLOSURES

No conflicts of interest, financial or otherwise, are declared by the author(s).

## AUTHOR CONTRIBUTIONS

C.S. conceived and designed research; J.R., H.G., G.R., R.T., A.W., and C.S. performed experiments; J.R., P.R., G.V.H., M.M., S.T.S., T.K., E.N., P.C., M.R., T.M., and C.S. analyzed data; J.R., P.R., H.G., S.T.S., T.K., E.N., P.C., M.R., E.F., and C.S., interpreted results of experiments; J.R. prepared figures; J.R., and C.S. drafted manuscript; J.R., P.R., H.G., G.R., R.T., G.v.H., M.M., M.K., G.S., J.P.G., S.T.S., T.K., E.N., P.C., T.M., E.F., and C.S., edited and revised manuscript; J.R., P.R., H.G., G.R., R.T., G.v.H., M.M., A.W., M.K., G.S., J.P.G., S.T.S., T.K., E.N., P.C., M.R., T.M., E.F., and C.S. approved final version of manuscript.
